# R-loops and small RNA regulatory interactions: mechanisms and clinical perspectives

**DOI:** 10.3389/fcell.2025.1727548

**Published:** 2025-12-17

**Authors:** Si-Yao Wang, Ya-Ni Liu, Si-Qiao Zhao, Wen Liu, Jin-Long Hu, Fan Yang, Sheng Wang

**Affiliations:** China Medical University, Shengjing Hospital of China Medical University, Shenyang, China

**Keywords:** R-loops, small RNAs, RNA-DNA hybrids, genomic instability, DNA repair, transposon silencing

## Abstract

An R-loop is a three-stranded nucleic acid structure that serves as a transcriptional intermediate, consisting of an RNA-DNA hybrid and a displaced single-stranded DNA (ssDNA). Small RNAs are RNA molecules shorter than 300 nucleotides that perform a wide range of essential functions within cells. Both R-loops and small RNAs are widely present in the genomes of prokaryotes and eukaryotes, where they play crucial roles in regulating gene expression, maintaining genomic stability, and facilitating DNA damage repair. Aberrant formation and accumulation of R-loops, coupled with dysregulation of small RNA pathways, can induce DNA damage and genomic instability, ultimately contributing to cellular senescence or cell death. Here, we discuss recent advances in understanding the crosstalk between R-loops and small RNAs, with a focus on their synergistic roles in maintaining genome stability and their therapeutic potential in oncology and neurodegeneration. We propose a novel model integrating R-loop dynamics with small RNA-mediated epigenetic regulation, supported by emerging clinical trial data.

## 1 Background

R-loops, three-stranded nucleic acid structures comprising an RNA-DNA hybrid and a displaced single-stranded DNA, are dynamically generated during transcription and play dual roles in genome regulation. While essential for processes like immunoglobulin class switching and mitochondrial DNA replication, aberrant R-loop accumulation triggers DNA damage, genomic instability, and cellular senescence. Small RNAs (e.g., miRNAs, siRNAs, piRNAs) are critical post-transcriptional regulators, yet their interplay with R-loops remains inadequately explored. Several key gaps persist:

1. A unified model integrating R-loop dynamics with small RNA biogenesis across cell types is lacking.
2. Neuron-specific regulatory mechanisms remain undefined.
3. Clinical translation is hindered by the blood-brain barrier (BBB) impermeability to small RNA therapeutics.

This review discusses recent advances in establishing a cross-scale regulatory framework, elucidating how R-loops and small RNAs cooperatively govern genomic stability. We also propose AI-driven hotspot prediction and BBB-penetrant nanocarriers as transformative strategies for neurological and oncological applications.

## 2 Introduction

An R-loop is a three-stranded nucleic acid structure that typically forms during transcription (Niehrs and Luke, 2020). It consists of an RNA-DNA hybrid duplex and a single-stranded DNA (Marabitti et al., 2022; Brickner et al., 2022; Lin et al., 2022; Petermann et al., 2022; Crossley et al., 2023; Elsakrmy and Cui, 2023; Xu et al., 2023; Yang et al., 2023). Specifically, an R-loop forms when the RNA molecule binds to one of the DNA template strands, creating an RNA-DNA hybrid, while the other DNA strand (the non-template strand) remains exposed as a single strand. Although R-loops are a natural phenomenon during transcription, excessive accumulation can lead to genomic instability, transcriptional errors, or DNA damage if not properly regulated (Pinto et al., 2024; Ren et al., 2024; Jin et al., 2023; Krishnan et al., 2023; Laspata et al., 2023). In addition to transcription, R-loops participate in various cellular processes such as DNA repair and transposon silencing, highlighting their multifunctional roles. Recently, research on R-loops has gained significant attention, particularly concerning their roles in maintaining genomic stability, regulating transcription, and responding to cellular stress.

Small RNAs are non-coding RNAs, ranging from 20 to 300 nucleotides in length, with crucial regulatory functions. They primarily regulate gene expression at the post-transcriptional level by pairing with target mRNA bases or interacting with target proteins (Wei et al., 2012; Xiong and Zhang, 2023). Based on their biological functions, lengths, and involvement in gene regulation, small RNAs can be categorized into several types, including microRNA (miRNAs), small interfering RNA (siRNA), Piwi-interacting RNA (piRNA), small nuclear RNA (snRNA), small nucleolar RNA (snoRNA), and tRNA-derived fragments (tsRNA). Each type of small RNA has distinct functions and mechanisms in the cell. Small RNAs are key regulatory factors in gene expression, genomic stability, and essential biological processes such as cell development and differentiation. Their mechanisms of action mainly involve interactions with target RNAs, and they also play important roles in various disease processes.

While previous reviews have focused on the biogenesis of R-loops or the functions of small RNAs separately, the mechanistic interplay between these two entities remains underexplored, especially in the context of cancer immunotherapy and age-related diseases. This study aims to elucidate the bidirectional regulatory mechanisms between R-loops and small RNAs and explore their potential applications in maintaining genomic stability and treating diseases.

## 3 Interactions between R-loop and small RNAs

In cells, the biosynthesis, function, and regulation of small RNAs involve complex post-transcriptional regulatory processes.

### 3.1 R-loops regulation of small RNA biosynthesis

**FIGURE 1:**
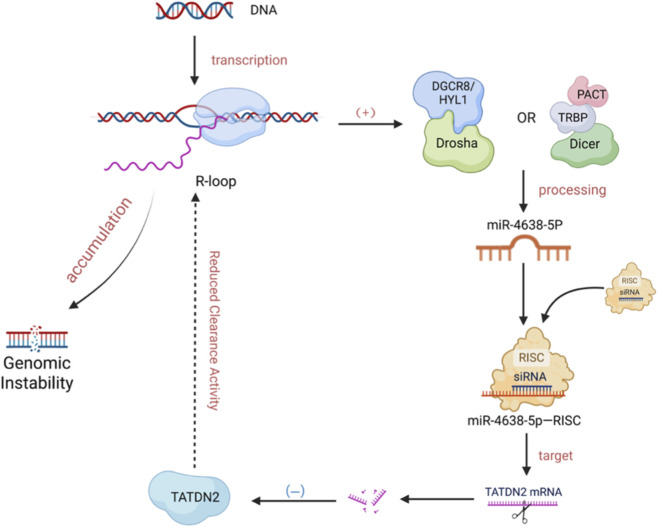
Bidirectional regulation of miRNA and R-loop. R-loops Facilitate miRNA Maturation. The formation of an R-loop (comprising an RNA-DNA hybrid and displaced single-stranded DNA) during transcription by RNA Polymerase II (RNAP II) promotes the co-transcriptional processing of primary miRNA (pri-miRNA) by the Drosha/Dicer complex, thereby enhancing the efficiency of mature miRNA production. miRNA Modulates R-loop Homeostasis. The mature miRNA, miR-4638-5p, binds to the 3′UTR of TATDN2 mRNA, inhibiting the expression of this R-loop-resolving enzyme. This suppression leads to the accumulation of unresolved R-loops, which subsequently contributes to genomic instability. (Created in BioRender. SY, W (2025) https://BioRender.com/8u9svd6).

R-loops directly regulate the transcription and maturation of small RNAs. For example, their presence at miRNA loci facilitates the co-transcriptional processing of pri-miRNAs into precursors, thereby enhancing mature miRNA production. Similarly, R-loops impact the transcription of piRNA precursors and can influence siRNA efficiency by altering target accessibility (Zhu et al., 2022). R-loops may also interfere with post-transcriptional splicing or other modification steps, thus impacting miRNA maturation. Additionally, small RNA biosynthesis usually requires specific enzymes to process the transcribed precursor RNAs (e.g., Drosha, Dicer, etc.) (Camino et al., 2023). R-loops may alter the structure of the transcript, influencing the cleavage sites and efficiency of Drosha and Dicer (As shown in Figure 1). For instance, R-loops may alter RNA’s spatial structure or provide additional protein binding sites, thereby modifying small RNA generation (Dettori et al., 2021; Thompson et al., 2023). Some studies suggest that R-loops may form complexes with specific RNA-binding proteins (e.g., PARN, CPSF, etc.) (Elsakrmy and Cui, 2023; Sanya et al., 2023), regulating processes such as splicing, capping, or polyadenylation of miRNAs precursor RNAs, all of which are critical steps in small RNA maturation.

### 3.2 R-loops and small RNA-mediated gene silencing

Small RNAs (such as siRNA and miRNA) mediate gene silencing by binding to target mRNA, activating RNA degradation, or inhibiting translation. Gene silencing by small RNAs primarily occurs through two mechanisms: post-transcriptional repression and mRNA degradation (Boichard et al., 2021; Pang et al., 2023; Vermunt et al., 2023; Ando et al., 2024). Small RNAs (especially miRNAs) can bind to the 3′untranslated region (3′UTR) of target mRNA to inhibit translation. R-loops may indirectly regulate miRNA target recognition and translation repression by influencing RNA structure during transcription. For example, R-loops may alter the secondary structure of mRNA, affecting small RNA binding to targets, regulating miRNA target selection, and influencing specific RNA structures to become targets for specific miRNAs. Some R-loops may also provide additional target regions for specific small RNAs, enhancing the specificity of gene silencing (Ginno et al., 2012; Bayona-Feliu et al., 2017). The accumulation of R-loops may affect the binding efficiency of small RNAs to their targets, thus modifying the gene silencing effect mediated by small RNAs.

### 3.3 R-loops and regulation of small RNA target genes

R-loops are closely associated with the regulation of gene expression, particularly in the transcriptional regulation of certain genes (Ginno et al., 2012). R-loops may provide a new regulatory mechanism for small RNA regulation. R-loops can impact mRNA degradation, especially in RNA interference (RNAi)-related degradation mechanisms (Elbashir et al., 2001). R-loops may interact with RNA degradation mechanisms (such as AGO protein complexes) to promote target mRNA degradation. The presence of R-loops may make mRNA more susceptible to recognition by degradation enzymes or accelerate its degradation by affecting mRNA stability. In RNA interference (RNAi), AGO proteins bind to small RNAs to form the RNA-induced silencing complex (RISC), which then guides target mRNA degradation (Wu et al., 2020). R-loops may facilitate small RNA target recognition and degradation through their potential interaction with AGO protein complexes, possibly enhancing the efficiency of RNA-induced silencing complex (RISC)-mediated degradation pathways.

## 4 Core interaction mechanism

### 4.1 Regulation of small RNA biogenesis and function by R-loops

R-loops act as dynamic genomic hubs that directly orchestrate the transcription, maturation, and functional output of diverse small non-coding RNAs, playing a central role in post-transcriptional regulatory networks.

#### 4.1.1 miRNAs

R-loops frequently form at miRNA genomic loci, promoting their biogenesis. They facilitate the co-transcriptional processing of primary miRNAs (pri-miRNAs) by the microprocessor complex (e.g., DROSHA/DCL1), enhancing the production of mature miRNAs (Zhu et al., 2022; Lambo et al., 2019). This effect is further modulated by R-loop-mediated changes in RNA Polymerase II (RNAPII) elongation dynamics (Gonzalo et al., 2022; Castel et al., 2014). Conversely, mature miRNAs can fine-tune R-loop homeostasis, establishing a feedback loop. For instance, miR-4638-5p suppresses TATDN2, an enzyme that resolves R-loops by degrading the RNA strand, leading to R-loop accumulation and genomic instability (Jaiswal et al., 2023). Similarly, miR-346 is correlated with R-loop formation, possibly through mechanisms such as stalling DNA replication or promoting transcription initiation upon chromatin binding initiation upon chromatin binding (Fletcher et al., 2022) (As shown in Figure 1).

#### 4.1.2 siRNAs

siRNA specifically targets mRNA and inhibits its expression through degradation. It utilizes its gene-silencing function to remove RNA-DNA helicases, thereby promoting R-loop accumulation. For example, in the case of human papillomavirus (HPV), SETX is a known helicase that can resolve R-loops. In the presence of siRNA, SETX depletion leads to increased R-loops, which interferes with viral transcription and enhances the integration of the viral genome (Jose et al., 2024). Similarly, siRNA can also affect R-loop stability by downregulating the reverse transcriptase ribonuclease H (RNase H). Specifically, RNase H enzymes are nucleases that hydrolyze the RNA portion of RNA: DNA hybrids, with two types of RNase H proteins: H1 and H2 (Cerritelli and Crouch, 2009). RNase H1 is active throughout the cell cycle, whereas RNase H2 is primarily active during the G2/M phase, where it removes R-loops (Lockhart et al., 2019). Furthermore, R-loop formation depends on three factors: high G-density, negative supercoiling, and DNA nicks (Tan-Wong et al., 2019). Relaxation of negative supercoiling can reduce R-loop formation, and DNA topoisomerase 1 (Top1) can achieve this by cleaving the DNA strand of RNA-DNA hybrids to form a transient Top1-DNA cleavage complex (Top1cc), which regulates the rotation of the cleaved strand around the uncut strand. Similarly, siRNA can silence Top1 to modulate the number of R-loops (Manzo et al., 2018). Thus, siRNA exerts its gene-silencing function by regulating enzymes related to R-loop formation, such as SETX, RNase H, and Top1. By removing or inhibiting these enzymes, siRNA induces R-loop accumulation, thereby affecting viral transcription and genomic integration. Moreover, the regulation of R-loop stability by siRNA provides new insights into the regulation of gene expression and genomic stability.

#### 4.1.3 piRNAs

The interaction between piRNAs and R-loops is multifaceted. piRNA precursor transcripts, synthesized by RNAP II, can accumulate R-loops during transcriptional elongation and termination, suggesting that R-loop dynamics influence piRNA biogenesis (Yu et al., 2021). More prominently, the piRNA-PIWI complex acts as a genome-stabilizing effector by directly resolving R-loops. PIWIL4, a PIWI protein with RNase H-like activity, binds piRNAs to form a complex that specifically degrades transposon-associated R-loops, preventing DNA damage (Bamezai et al., 2023).

Beyond the canonical small non-coding RNAs, the functional interplay between R-loops and RNA extends to include longer non-coding transcripts and small nuclear RNAs. Although long non-coding RNAs (lncRNAs, typically >200 nucleotides) fall outside the standard definition of small RNAs, their interactions with R-loops are mechanistically profound and frequently involve the formation of R-loop structures as a core component of their function (Nojima et al., 2018). Similarly, small nuclear RNAs (snRNAs), while categorized as small RNAs, exert their influence on R-loop homeostasis primarily through an indirect, functional association with the spliceosome. The following sections will explore the distinct mechanisms by which these two important RNA classes—lncRNAs and snRNAs—interface with R-loop biology.

#### 4.1.4 lncRNAs

For long non-coding RNAs, R-loops are often integral to their functional mechanisms. Certain lncRNAs, such as APOLO and TARID, form R-loops at specific genomic loci via complementary base-pairing, regulating transcription of nearby genes (Ariel et al., 2020; Arab et al., 2019). A canonical example is the telomeric lncRNA TERRA, whose G-rich sequence readily forms R-loops with the C-rich telomeric strand. These structures are crucial for telomere length regulation and chromosome end protection, particularly in the Alternative Lengthening of Telomeres (ALT) pathway (Vohhodina et al., 2021; Yadav et al., 2022; Vaid et al., 2024). Additionally, R-loops can actively promote lncRNA synthesis: the exposed single-stranded DNA within R-loops may act as a promoter, driving antisense transcription and facilitating lncRNA production (Tan-Wong et al., 2019).

#### 4.1.5 snRNAs

For small nuclear RNAs (snRNAs), while no direct interaction with R-loops has been established, an important indirect functional link exists through the spliceosome. snRNAs (e.g., U1, U2) are core components of the spliceosome, and splicing factors like XAB2 and LUC7L3, which associate with snRNPs, play a key role in maintaining R-loop homeostasis (Goulielmaki et al., 2021; Zhang X. et al., 2024). Depletion of these factors leads to R-loop accumulation, especially following DNA damage or transcriptional stress, when they are released from the spliceosomal complex (Jurica and Moore, 2003). This underscores the importance of snRNA-mediated splicing machinery in maintaining cellular processes and its indirect role in R-loop regulation and prevent their accumulation under certain conditions.

In summary, R-loops serve as a fundamental regulatory layer in the life cycle of small non-coding RNAs. They act as platforms that enhance the biogenesis of miRNAs and piRNAs, serve as structural elements for lncRNA function, and their homeostasis is indirectly safeguarded through the snRNA-spliceosome axis. This intricate crosstalk highlights the pivotal role of R-loops in integrating transcription with RNA-mediated regulation.

### 4.2 Regulation of R-loop homeostasis by small RNAs

Small RNAs precisely regulate R-loop dynamics through distinct strategies, controlling R-loop accumulation and resolution. This regulation plays a pivotal role in critical cellular processes, including antiviral defense and transposon silencing.

**FIGURE 2:**
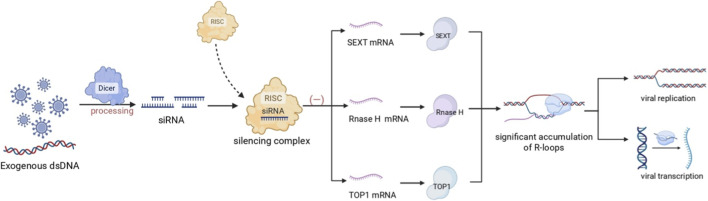
Current small RNA-based R-loop targeted therapy. Exogenous double-stranded RNA (dsRNA), such as from a viral infection, is processed by Dicer into siRNAs. These siRNAs are loaded into the RNA-induced silencing complex (RISC) and guide it to silence the mRNAs of key R-loop-resolving enzymes, including the helicase SETX, Ribonuclease H (RNase H), and DNA Topoisomerase 1 (TOP1). The coordinated depletion of these enzymes results in R-loop accumulation through reduced RNA degradation, impaired hybrid unwinding, and the persistence of negative supercoiling. This elevated R-loop burden ultimately inhibits viral transcription, constituting an intrinsic host defense mechanism. (Created in BioRender. SY, W (2025) https://BioRender.com/9jcbub0).

siRNAs fine-tune R-loop stability through a post-transcriptional mechanism. By leveraging their sequence-specific gene silencing capability, siRNAs can target and deplete key enzymes responsible for resolving R-loops. A prime example is seen during viral infection, where siRNA-mediated silencing of the R-loop helicase SETX intentionally induces R-loop accumulation. This accumulation disrupts human papillomavirus (HPV) transcription and genome integration (Jose et al., 2024). Similarly, siRNAs can target core resolvers like Ribonuclease H (RNase H), which degrades the RNA strand in RNA-DNA hybrids, and DNA topoisomerase 1 (Top1), which alleviates negative supercoiling to prevent R-loop formation (Jose et al., 2024; Tan-Wong et al., 2019). The coordinated suppression of these factors (SETX, RNase H, Top1) via the RNAi pathway serves as a potent mechanism to manipulate R-loop levels, facilitating antiviral defense and providing new insights into gene regulation. (As shown in Figure 2).

The piRNA system regulates R-loop clearance in the germline through both direct and indirect pathways. Piwi-interacting RNAs (piRNAs) guide PIWI proteins, such as PIWIL4, which possesses RNase H-like activity, to directly target and resolve R-loops at transposons loci. This process prevents DNA damage and ensures genomic integrity (Suzuki et al., 2023; Schmitz et al., 2022). In addition, the piRNA-PIWI complex acts as a master regulator of transposon silencing by recruiting epigenetic modifiers to establish repressive heterochromatin at transposon loci. This transcriptional repression reduces the production of nascent transcripts, which are prone to form R-loops (Fabry et al., 2021; Huang et al., 2017; Parikh et al., 2018). The combined strategies of direct resolution and transcriptional silencing form a positive feedback loop, effectively constraining transposon activity and maintaining R-loop homeostasis.

In summary, small RNAs act as central commanders of R-loop equilibrium. siRNAs target the R-loop resolution machinery for post-transcriptional degradation, while piRNAs deploy a multifaceted strategy involving both direct enzymatic cleavage and epigenetic silencing. This sophisticated small RNA-mediated regulation is fundamental to cellular functions, including genome defense and maintaining genomic stability across generations.

### 4.3 The epigenetic state of chromatin as a key determinant of R-loop formation

The epigenetic state of chromatin is crucial for regulating R-loop formation. Open chromatin regions that are rich in active histone marks—such as those found in promoter regions and gene bodies—are transcriptionally active and serve as hotspots for R-loop formation (Fabry et al., 2021). In contrast, regions of heterochromatin, marked by repressive histone modifications, such as H3K9me3 and H3K27me3, exhibit transcriptional suppression, which leads to a reduction in R-loop formation (Guo et al., 2021). Notably, this regulation is bidirectional: R-loops themselves can also serve as platforms to recruit epigenetic modifiers, altering the local chromatin state. Together, these processes form a dynamic feedback loop that finely tunes gene expression and contributes to genome stability.

### 4.4 The R-loop-small RNA axis in genome stability

The R-loop-small RNA axis constitutes a critical, self-reinforcing regulatory module that safeguards genomic integrity through a series of bidirectional feedback mechanisms. This coordination operates at multiple levels, spanning RNA processing to epigenetic regulation.

Firstly, R-loops directly influence the biogenesis and stability of small RNAs, thereby shaping the cell’s regulatory capacity. As structures that emerge during transcription, R-loops can affect the efficiency of small RNA precursor synthesis by causing transcriptional stalling, which disrupts the production of primary transcripts, such as pri-miRNAs (Cornec and Poirier, 2023; Jimeno et al., 2021). Furthermore, R-loops can interfere with subsequent nuclear and cytoplasmic maturation steps, impacting the cleavage efficiency of processing enzymes like Drosha and Dicer, ultimately altering the yield of mature, functional small RNAs (Camino et al., 2023; Fabry et al., 2021; Huang et al., 2017; Parikh et al., 2018). Beyond their role in biogenesis, R-loop accumulation can create a hostile environment for small RNAs, potentially promoting their degradation via nucleases or interfering with their function, thus impairing the post-transcriptional regulatory network (Boque-Sastre et al., 2015; Kaneko et al., 2007).

Conversely, small RNAs and their associated factors actively maintain R-loop homeostasis to prevent DNA damage. This is achieved through both direct and indirect mechanisms. The RNA interference (RNAi) machinery, guided by siRNAs or miRNAs, can silence the expression of key R-loop resolvers, such as the helicase SETX, achieving specific biological outcomes (Lundstrom, 2020; Appel et al., 2023). More broadly, cells utilize transcription factors and epitranscriptomic mechanisms to dynamically control R-loop levels. For instance, the stress-responsive transcription factor TonEBP can recognize R-loop structures and recruit the m6A writer METTL3. This METTL3-mediated deposition of N6-methyladenosine (m6A) on R-loops facilitates their resolution, representing a crucial epitranscriptomic pathway for maintaining genomic stability (Kang et al., 2021a). Other factors, such as the co-regulator Thrap3, help reduce R-loop accumulation by recruiting helicases like DDX5 (Kang et al., 2021b; Wen et al., 2024). Conversely, the loss of transcription elongation factors like SPT6 can disrupt normal transcription and lead to pathogenic R-loop accumulation, linking this axis to processes like cellular aging (Zhang J. et al., 2024).

In summary, the R-loop-small RNA axis forms a robust, self-correcting circuit. R-loops shape the small RNA landscape, which, through RNAi and the recruitment of enzymatic resolvers, fine-tunes R-loop dynamics. This intricate interplay, coupled with transcription-coupled resolution mechanisms, is indispensable for preventing transcription-replication conflicts, mitigating DNA damage, and ensuring long-term genomic stability.

### 4.5 Cross-species perspective: conservation and specificity of R-loops and small RNA interactions in prokaryotes and plants

#### 4.5.1 Conservation and specificity in prokaryotes

In bacteria, R-loops also represent a significant source of genomic instability. Small RNAs (sRNAs) play a central role in prokaryotic gene expression regulation (Pandiyan et al., 2024). A prominent example of “small RNA-guided DNA recognition” in prokaryotes is the CRISPR-Cas system. In this system, CRISPR RNAs (crRNAs) guide Cas proteins to target DNA via sequence complementarity, forming transient RNA-DNA hybrids (like R-loops), which are essential for DNA cleavage (Pacesa et al., 2022; Vlachos-Breton and Drolet, 2022; Cai et al., 2025). This mechanism can be viewed as a strictly regulated, defense-related form of R-loop formation.

Unlike eukaryotes, bacteria lack the typical RNA interference (RNAi) machinery, such as Dicer and AGO proteins. As a result, sRNAs in bacteria regulate mRNA stability and translation primarily through interactions with proteins (e.g., Hfq), rather than by forming RNA-induced silencing complexes (RISC) to degrade RNA-DNA hybrids (Xu et al., 2024). Therefore, R-loop regulation in prokaryotes is likely more reliant on enzymes like RNase H, which degrade RNA-DNA hybrids directly.

#### 4.5.2 Conservation and specificity in plants

Plants, like animals, possess a comprehensive suite of miRNAs, siRNA, and piRNA pathways that contribute to genomic stability and transposon silencing. R-loop formation can also influence these processes in plants (Zheng et al., 2023; Zhou et al., 2022). Additionally, plants feature a unique 24-nt siRNA-mediated RNA-directed DNA methylation (RdDM) pathway, where siRNAs guide DNA methyltransferases to homologous genomic regions (Gao et al., 2021). Research has shown that these regions are particularly susceptible to R-loop formation, with the generation of R-loops either promoting or relying on the RdDM pathway (Fonouni-Farde et al., 2022). Together, these mechanisms establish and maintain transcriptional silencing and epigenetic stability.

As sessile organisms, plants face distinct environmental pressures, including UV radiation and pathogen attacks. The interaction between R-loops and small RNAs may play a special role in how plants respond to these biotic and abiotic stresses, adding an additional layer of complexity to their regulatory networks. A summary of the functional divergence of different small RNA types in regulating R-loop dynamics is provided in Table 1.

**TABLE 1:** Functional divergence of small RNAs in R-loops regulation. This table systematically summarizes the distinct roles and mechanisms of different small RNA types in regulating R-loop dynamics and their associated pathogenic outcomes.

Small RNA type	Primary target	Regulatory effect on R-loop	Mechanistic link to pathogenesis	Associated diseases
miRNA	*TATDN2, DICER1*	Inhibits degradation →R-loop accumulation↑	Genomic instability from unresolved R-loops drives oncogenesis R-loop-driven transcription disrupts neurodevelopmental gene networks	Ovarian cancer, autism spectrum disorder
siRNA	*SETX, RNase H*	Silences helicases →R-loop stability↑	Persistent R-loops hinder viral transcription/integration (e.g., HPV) or induce transcription-associated DNA damage in neurons	HPV infection, neurodegenerative diseases
piRNA	*PIWIL4, Transposon-derived RNA*	Recruits degradation complexes →R-loop↓	Failure to silence retrotransposons leads to insertional mutagenesis and genomic rearrangements in rapidly dividing or germline cells	Leukemia, disorders of germ cell development
LncRNA	*Promoter regions*	Directly forms R-loop → transcriptional regulation	Aberrant transcription of oncogenes/tumor suppressors. Dysregulated telomere maintenance via the ALT pathway promotes cellular immortalization	Prostate cancer, telomere lengthening (ALT pathway)

## 5 Clinical applications and prospects

As the mechanisms underlying the interaction between R-loops and small RNAs continue to be better understood, the potential of this field in clinical and disease treatment is becoming increasingly evident. Particularly in cancer, neurodegenerative diseases, aging, and genetic disorders, it holds significant promise for future applications.

### 5.1 Cancer treatment

Genomic instability is commonly observed in cancer cells, and the accumulation of R-loops is considered a key factor contributing to genomic instability, DNA damage, and mutations (Xu et al., 2023; Zong et al., 2023; Castillo-Guzman and Chédin, 2021). Excessive accumulation of R-loops can result in transcription stalling, DNA damage, and chromosomal abnormalities. Therefore, regulating the formation and removal of R-loops could represent a novel approach in cancer therapy.

**FIGURE 3:**
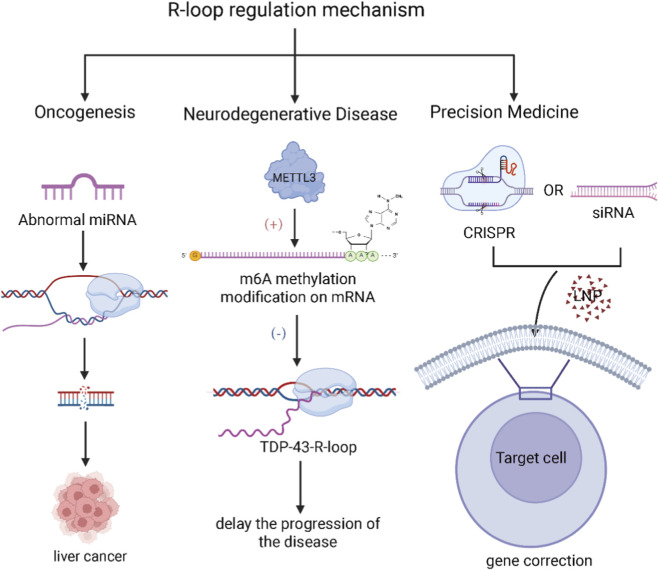
Proposed small RNA-based strategies targeting R-loop pathology (Left) Cancer: Dysregulated miRNA expression can influence R-loop levels, thereby promoting oncogenesis and tumor progression. (Center) Neurodegenerative Diseases: The accumulation of TDP-43-R-loops is a hallmark of certain neurodegenerative conditions. METTL3-mediated m6A modification has been shown to resolve these pathogenic R-loops, potentially delaying disease onset and progression. (Right) Gene Therapy: Emerging therapeutic strategies include the use of lipid nanoparticles (LNPs) to deliver small RNA-based therapeutics, as well as CRISPR-based gene editing systems designed to precisely correct R-loop-associated mutations in affected genes. (Created in BioRender. SY, W (2025) https://BioRender.com/u64e5wp).

Regulatory Role of Small RNAs: Small RNAs, such as miRNAs and siRNAs, can regulate R-loops-related proteins, influencing the formation, stability, and elimination of R-loops (Wang et al., 2024). For instance, miRNAs may target specific transcription factors or DNA repair factors, indirectly modulating R-loop homeostasis and reducing genomic instability. Therefore, modulating these pathways via small RNAs represents a potential direction for future cancer research and therapeutic exploration (Molfino et al., 2023; Sun et al., 2019) (As shown in Figure 3).

RNA-targeted therapy aims to design specific small RNAs, which could potentially intervene in the accumulation of R-loops or promote their unwinding, helping cancer cells maintain genomic stability and prevent carcinogenesis (Vaid et al., 2024; Toden et al., 2021; Cao et al., 2022). Additionally, certain tumor types exhibit R-loop signatures that can serve as targets, allowing small RNAs or small molecule inhibitors to regulate their formation and restrict tumor growth.

### 5.2 Neurodegenerative diseases

Neurodegenerative diseases (e.g., Alzheimer’s disease, Huntington’s disease, amyotrophic lateral sclerosis) are closely linked to genomic instability, defects in DNA damage repair, and RNA aggregation (Wu et al., 2023). Abnormal accumulation of R-loops has been shown to exacerbate the onset and progression of these diseases.

Role of Small RNAs in R-loops Elimination: Small RNAs, particularly miRNAs, can regulate DNA repair pathways, reduce R-loops stability, and thereby decrease DNA damage (Sener et al., 2024). For example, certain small RNAs can target and inhibit the expression of proteins involved in R-loop resolution (such as RNase H), and modulating these pathways has been shown to promote R-loop removal and slow disease progression in models of neurodegeneration (Sanya et al., 2023; D'Alessandro et al., 2018). Future therapeutic strategies could therefore focus on targeting R-loop accumulation, including the design of specific small RNAs or small molecules to enhance R-loops unwinding and clearance. These strategies hold the potential not only to alleviate symptoms but also to delay the progression of neurodegenerative diseases.

### 5.3 Aging and genome stability

Aging is closely associated with the decline in DNA damage and repair mechanisms. As individuals age, the accumulation of R-loops increases, leading to genomic instability and the development of age-related diseases.

Intervention by Small RNAs: By regulating the expression of small RNAs, particularly those involved in DNA repair and R-loops clearance, it may be possible to help maintain genome stability and slow the aging process. Small RNAs could target proteins that promote R-loops clearance (e.g., SMARCAL1, RNase H) to repair DNA damage caused by R-loops (Bayona-Feliu et al., 2021).

Aging Intervention: Enhancing the functionality of DNA repair pathways through small RNA modulation could serve as a therapeutic strategy for anti-aging. As the potential of small RNAs in age-related diseases is further explored, this field may bring new anti-aging therapies in the future.

### 5.4 Genetic diseases and gene repair

In many genetic disorders, gene mutations and the accumulation of DNA damage result in abnormal cellular function. The formation and accumulation of R-loops may be closely linked to the onset of these diseases, particularly when gene mutations affect the transcription process.

Small RNA-mediated Gene Editing: Small RNAs could regulate the formation and elimination of R-loops in combination with gene editing technologies like CRISPR/Cas9, enabling the repair or correction of mutations in the genome and restoring normal gene expression (Wang et al., 2022; Bak et al., 2021). For example, small RNAs may adjust the transcriptional activity of specific genes to reduce mutations and genomic instability caused by R-loops, thus treating genetic diseases.

Precision Medicine: By precisely controlling R-loop formation using small RNAs, interventions could be developed for specific genetic diseases. For instance, targeting R-loop accumulation caused by specific genetic mutations and regulating R-loops clearance with small RNAs or small molecules could provide a new approach for treating genetic disorders.

### 5.5 Challenges and prospects in clinical treatment

Despite significant progress in the basic research of R-loops and small RNA interactions, several challenges remain for clinical applications, primarily regarding specificity and efficacy, delivery systems, and potential drug side effects and safety concerns. The specificity and effectiveness of small RNAs as therapeutic tools still need further validation. Ensuring that small RNAs precisely target specific R-loops regions without interfering with other crucial cellular functions will be a key focus for future research.

RNA Delivery Challenges: The delivery of small RNAs remains a significant hurdle. Developing efficient and safe delivery systems to ensure small RNAs effectively reach the target cells and perform their intended functions is a critical task for clinical applications.

Side Effects: Potential side effects, such as off-target activity, must be thoroughly investigated to ensure the safety and feasibility of clinical applications of small RNAs. Minimizing adverse effects while maximizing therapeutic potential will be essential for the successful use of small RNAs in clinical settings.

## 6 Physiological and pathophysiological outputs of the R-loop–small RNA network

The intricate interplay between R-loops and small RNAs extends beyond fundamental housekeeping roles, exerting critical influence over a broad range of physiological and pathophysiological processes.

### 6.1 Regulation of development and cellular differentiation

The R-loop–small RNA network is instrumental in executing complex developmental programs and guiding cellular differentiation. During development, precise temporal and spatial gene expression patterns are paramount. R-loops can influence the transcription of key developmental genes, while small RNAs, particularly miRNAs, provide a robust post-transcriptional layer of control that fine-tune protein output from these genes (Bayona-Feliu et al., 2021). This coordination ensures the precise transitions between developmental stages and the establishment of specific cell fates. Dysregulation of this network can lead to developmental defects and compromised tissue homeostasis.

### 6.2 Roles in metabolism and metabolic adaptation

Cellular metabolism is closely linked to gene expression networks, and the R-loop–small RNA axis serves as a key interface. Small RNAs can rapidly modulate the expression of metabolic enzymes and transporters in response to nutrient availability or energy stress (Heuzé et al., 2023; Liu et al., 2024). Concurrently, metabolic shifts can alter the transcriptional landscape and consequently affect the R-loop profile, creating a feedback loop that enables the cell to adapt its metabolic output. This positions the R-loop–small RNA network as a dynamic regulator of metabolic flux and energy balance.

### 6.3 Orchestration of immune and inflammatory responses

The R-loop–small RNA network plays a pivotal role in innate immunity and inflammation (Berkhout, 2018; Kong et al., 2023; Li et al., 2025). The recognition of certain R-loop structures or their byproducts can itself trigger innate immune signaling pathways. Furthermore, small RNAs, such as siRNAs and miRNAs, are central to antiviral defense—both by directly targeting viral transcripts and by regulating the expression of host immune factors. Dysregulated accumulation of R-loops or aberrant small RNA expression can contribute to chronic inflammatory diseases and autoimmune conditions.

### 6.4 Impact on cellular senescence and aging

The decline in cellular homeostasis during aging is closely linked to the R-loop–small RNA axis. The accumulation of R-loops is both a cause and a consequence of cellular senescence, a key driver of aging. Age-related dysregulation of small RNA biogenesis and function impairs the cell’s ability to maintain R-loop homeostasis and repair age-associated DNA damage, creating a vicious cycle that accelerates the functional decline of tissues and organisms (Fariyike et al., 2019).

## 7 Conclusions

This review summarizes key insights into the R-loop-small RNA network: 1. R-loop-Mediated Small RNA Biogenesis, R-loop-induced alterations in RNA secondary structures, such as G-quadruplex formation, can influence Drosha/Dicer processing of pre-miRNAs/pre-siRNA, thereby modulating downstream gene silencing. 2. piRNA-PIWI Complexes Maintain Genomic Integrity, in germ cells, piRNAs guide PIWI proteins to regulate transposon-linked R-loops, helping to protect genomic stability and prevent chromosomal fragmentation. This mechanism is relevant to fertility and may have implications for early-onset neoplasia. 3. Therapeutic Potential of R-loop Modulation: Experimental studies suggest that strategies to control R-loop accumulation, including small RNA–or CRISPR-based approaches, may offer neuroprotective effects in models of neurodegenerative diseases such as Alzheimer’s. While promising, these findings require further validation before clinical translation. Building on these insights, future research should focus on: 1. Targeted Delivery Systems: Developing nanocarriers capable of delivering small RNAs specifically to the CNS or other tissues of interest. 2. Predictive Platforms: Integrating high-resolution R-loop mapping with computational models to identify pathogenic R-loop/small RNA interaction hotspots. 3. Mechanistic Studies in Disease Contexts: Using advanced models, such as cerebral organoids, to track R-loop dynamics and their causal roles in neurodegeneration and tauopathy. In summary, the crosstalk between R-loops and small RNAs constitutes a fundamental regulatory axis that governs genomic stability, precise gene expression, and cellular stress responses. A deeper understanding of these interactions could reveal novel therapeutic avenues for oncology, neurodegeneration, and aging-related diseases. Future advancements in targeted delivery systems and AI-driven predictive models will be pivotal in translating these mechanistic insights into clinical applications.
